# Evaluation of Isosorbide Mononitrate for Preinduction of Cervical Ripening: A Randomized Placebo-Controlled Trial

**Published:** 2015-06

**Authors:** Ramya Krishnamurthy, P. Pallavee, Seetesh Ghose

**Affiliations:** Department of Obstetrics and Gynaecology, Mahatma Gandhi Medical College & Research Institute, Pondicherry, India

**Keywords:** Isosorbide mononitrate, cervical ripening, preinduction, placebo

## Abstract

**Objective:**To evaluate the safety and efficacy of Isosorbide mononitrate (IMN) as a cervical ripening agent prior to induction of labour in term pregnant women.

**Materials and methods:**A randomized placebo-controlled study was conducted on 100 term singleton pregnancies planned for induction of labour. The participants were randomly assigned to two groups. One group received 40 mg IMN and the other group received 40mg of placebo kept vaginally. The main outcome of this study was to evaluate the efficacy of IMN in cervical ripening based on the change in modified Bishop score and the effect on time duration between the drug insertion and delivery. Safety of isosorbide mononitrate was assessed by measuring variables related to maternal and neonatal outcomes.

**Results:**Baseline demographic characteristics were similar in both groups. The mean change in modified Bishop score after 2 doses of 40mg IMN was insignificant when compared to placebo. Though IMN shortened the time duration between the drug insertion to delivery when compared to placebo, it was statistically insignificant. The need for oxytocin and 2^nd^ ripening agent was less in IMN group when compared to placebo group but statistically this also proved to be insignificant. It was noted that there was an increase in caesarean deliveries in IMN than in placebo group. IMN did not cause any significant change in maternal hemodynamics and adverse side effects. Though NICU admission and stay was less in IMN than in placebo group, it was statistically insignificant.

**Conclusion:**Though IMN did not cause any maternal and neonatal adverse effects, it was found to be inefficient in comparison to placebo as a cervical ripening agent.

## Introduction

Induction of labour is one of the most common interventions carried out in modern obstetrics. Induction implies stimulation of contractions before the spontaneous onset of labour, with or without rupture of membranes (1). The condition or favourability of cervix is an important prognostic indicator for the success of induction. In primigravida, the mean time taken from induction to delivery is 27 hours, of which up to 18 hours is spent in the cervical ripening phase before labour itself starts.Cervical ripening is an active process involving remodelling of cervical tissue which is crucial to the process of human parturition. It is an important conditioning step done mainly to reduce the risk of operative delivery and failure of induction (2). The ideal agent for cervical ripening would induce adequate cervical ripening without causing uterine contractions, uterine hypertonus and without the need for fetal monitoring with minimal maternal and fetal side effects. One of the major problems of using prostaglandins for cervical ripening is the induction of uterine contractions due to their stimulatory effect on the myometrium which sometimes requires tocolytic therapy (3). Human cervix is capable of producing nitric oxide and it has been postulated that nitric oxide represents the final metabolic mediator of cervical ripening at the end of the ripening cascade prior to onset of labour (3). Nitric oxide donors which liberate nitric oxide in vivo have generated an interest for use as compared to prostaglandins for cervical ripening because of their negligible uterine effects. In this study we aimed to evaluate the safety and efficacy of isosorbide mononitrate as a cervical ripening agent in term pregnant women prior to induction of labour.

## Materials and methods

This randomized controlled trial was conducted on 100 term pregnant women who were admitted for induction of labour and were approached for consent of participation. We included primigravida with singleton pregnancy and cephalic presentation, at gestational age of 38 completed weeks or more and with a modified Bishop score of less than 6. We excluded teenage (<18 yr) pregnancy, scarred uterus, ruptured membranes, presence of uterine contractions, medical complications, any contraindications to vaginal delivery and contraindications to Isosorbide mononitrate therapy. A written informed consent was taken from all the participants who fulfilled the inclusion and exclusion criteria of the study. Randomization of the participants into two groups was done according to a computer generated random number table and allocation was done according to sequentially numbered opaque envelope technique. One group received 40mg of isosorbide mononitrate (ISMO 40) and the other group received 40 mg of the placebo (Pyridoxine). The drug insertion was done by a senior resident who was not a part of the investigation. On admission, cervical assessment was done to see dilatation (cm), length (cm), position, consistency, and station of presenting part to get the modified Bishop score, the allocated drug was inserted vaginally and kept in the posterior fornix. 12hr later cervical assessment was repeated. If Bishop Score was<6, A second dose of treatment drug was given. For those whose cervical score remained<6, even after 24 hours from the first dose, a second ripening agent (PGE2 gel), was instilled intracervically for a maximum of 2 doses 6 hours apart. Those with favourable cervix (Bishop Score≥6) during the cervical ripening process underwent amniotomy along with oxytocin stimulation. Women who went into labour during the cervical ripening process were managed according to our hospital active management of labour protocol. Failure of induction was defined by non-occurrence of labour despite two doses of second ripening agent i.e., PGE2and where oxytocin stimulation using maximum effective dosage was done. Monitoring consisted of fetal cardiotocogram (FCTG), maternal pulse and blood pressure at 30 minutes before drug insertion then at 1hour and 6 hour post drug insertion. ACOG guidelines were followed for intrapartum monitoring of fetal heart rate and maternal vitals. The primary outcomes studied were the change in modified Bishop Score at 12 and 24 hr after drug insertion and the time from initiation of cervical ripening till delivery. We also studied secondary outcomes such as the delivery outcomes such as labour duration, need of oxytocin augmentation and mode of delivery. Maternal outcomes like uterine hyperstimulation, tachysystole, headache, tachycardia, palpitations, hypotension, nausea and vomiting were also recorded and compared between the groups. Fetal outcomes including Apgar scores < 7 at 1 min and 5 min, fetal distress, neonatal intensive care unit (NICU) admissions and length of neonatal stay in NICU were noted. We also compared the proportions of unripe cervix (Bishop Score < 6) at 24 hr after first drug insertion.

Descriptive and inferential statistical analyses havebeen carried out in the present study. Results of continuous measurements are presented as Mean ±SD and results of categorical measurements are presented as Number (%). Significance was assessed at 5% level. The following assumptions about the data were made: 1.Dependent variables are normally distributed, 2.Samples drawn from the population was random. Independent Student’s t test was used to find the significance of study parameters on continuous scale between two groups on metric parameters. Chi-square/ Fisher Exact test were used to find the significance of study parameters on categorical scale between groups. A P value of < 0.05 was considered statistically significant. Microsoft word and Excel were used to generate graphs, tablesetc**.**Statistical analysis was carried out using SPSS version 19.0 (IBM SPSS, US) software with regression modules installed.

## Results

The baseline characteristics of both the IMN and Placebo groups were similar. The mean age of patients in IMN and Placebo were 23.3±2.79 and 23.58±3.0year respectively. The mean gestational ages for the two groups were 40.26±0.85weeks.For IMN and 40.22±0.84weeks for placebo group. Majority of patients were induced for prolonged pregnancy (76% in IMN and 66% in placebo). The second most common indication was oligohydramnios and only one patient in IMN group was induced for Rh negative pregnancy at 40 wks. All the patients in both groups had a baseline modified bishop score ≤ 3, the mean being 1.78 ± 0.52 in IMN and and1.89 ± 0.38 in placebo group (Table 1).

There was no significant difference (p= 0.283) between the mean baseline cervical Bishop score for IMN (1.82± 0.52) and placebo (1.92± 0.38). The mean Bishop Score after 12 hours of drug insertion was 4.28± 1.01 and 4.41± 0.86 in IMN and placebo groups respectively. Similarly the mean scores were 4.64±1.33 in IMN and 4.77± 1.20 in placebo group at 24 hr post drug insertion (2 doses). There was no significant statistical difference in the change of mean cervical bishop score at 12hr, (mean difference -0.03, 95% confidence intervals -0.36 to 0.42, p = 0.881) and 24 hours (mean difference -0.03, 95% confidence intervals -0.53 to 0.54, p=0.983) between the two groups (Table 2).The mean time taken from drug insertion to delivery in IMN group and placebo group was 1787.30± 260.28 min and 1821.50± 269.25 mins respectively. IMN did not significantly shorten the time from drug insertion to delivery with the mean difference IMN- placebo being -34.2 (95% confidence interval -139.3 to 70.9, p=0.52).

Spontaneous vaginal delivery was seen more commonly in placebo group (70%) than in IMN group (48%) which was statistically significant (p= 0.025). Instrumental vaginal delivery was more common in IMN group than placebo with the mean difference being 4.26 (95% CI of 0.46 to 39.54, p= 0.02). Though, the incidence of caesarean section was more in IMN group than in placebo group, the difference did not reach statistical significance. There was no significant difference seen in length of labour, need of oxytocin and need for 2^nd^ ripening agent between the groups. 40 women in the IMN group and 40 women in placebo group had Bishop score less than 6 at the end of 24 hours from drug initiation. The proportions of patients with unripe cervix after 24 hours was similar in both the groups (80%) which was statistically insignificant (p= 1.000) (Table 3).

None of the patients in either of the groups had abnormal fetal heart tracings in CTG during the first 24 hrs. of drug insertion. There was no case of hyper stimulation of uterus or tachysystole during that period. No patient in IMN group complained of any side effects like headache, nausea, vomiting or palpitations. Maternal monitoring of pulse rate and blood pressure across the time points as described in the methodology did not reveal any instance of hypotension or tachycardia in the mother. IMN was found to be safe for use as a cervical ripening agent for both the mother and fetus.

**Table 1:** Baseline characteristics

Characteristics	IMN (n=50)	PLACEBO (n=50)
**Mean age in years±SD**	**23.3±2.79**	**23.58±3.0**
**Mean gestational age in weeks±SD**	**40.26±0.85**	**40.22±0.84**
**Indication of labour**		
** Prolonged pregnancy**	**38(76.0%)**	**33(66.0%)**
** Oligohydramnios**	**11 (22.0%)**	**17 (34.0%)**
** Rh negative pregnancy**	**1(2.0%)**	**0(0.0%)**
**Baseline modified Bishop score**		
** Mean±SD**	**1.78 ± 0.52**	**1.89±0.38**
** 1**	**12 (24.0%)**	**6 (12.0%)**
** 2**	**35 (70.0%)**	**42(84.0%)**
** 3**	**3 (6.0%)**	**2 (4.0%)**
** 0-3**	**50(100.0%)**	**50(100.0%)**

**Table 2:** Change in modified Bishop Score

MBS	IMN		PLACEBO		IMN-PLACEBO Mean difference(95% CI)	P value
	n	Mean	n	Mean		
**Baseline**	**50**	**1.82±0.52**	**50**	**1.92±0.40**	**-0.10(-0.28,0.08)**	**0.283**
**12 hours**	**50**	**4.28±1.01**	**49**	**4.41±0.86**	**-0.13(-0.50,0.25)**	**0.500**
**24 hours**	**45**	**4.64±1.33**	**47**	**4.77±1.20**	**-0.13(-0.65,0.40)**	**0.647**
**Mean change at 12 hours**	**50**	**-2.46±1.03**	**49**	**-2.49±0.94**	**-0.03(-0.36,0.42)**	**0.881**
**Mean change at 24 hours**	**45**	**-2.86±1.31**	**47**	**-2.87±1.26**	**-0.03(-0.53,0.54)**	**0.983**

**Table 3:** Labour and delivery outcomes

OUTCOME	IMN n=50	PLACEBO n=50	IMN-PLACEBO	P value
			Mean difference or Odds ratio(95% CI)	
**Mode of delivery**				
**Spontaneous Vaginal delivery**	**24(48.0%)**	**35(70.0%)**	**0.3956 (0.17, 0.89)**	**0.025**
**Instrumental vaginal delivery**	**4(8.0%)**	**1 (2.0%)**	**4.26 (0.46, 39.54)**	**0.020**
**Caesarean section**	**22 (44.0%)**	**14(28.0%)**	**2.02 (0.88, 4.64)**	**0.098**
**Need for oxytocin**	**44(88.0%)**	**47 (94.0%)**	**0.468 (0.110, 1.987)**	**0.303**
**Need for 2** ^nd^ ** RA** *	**42 (84.0%)**	**44 (88.0%)**	**1.397(0.447,4.367)**	**0.564**
**Mean length of labour ± SD(minutes)**	**440.52±145.58**	**434.18±139.88**	**6.34 (-51.2, 63.9)**	**0.827**
**Bishop score (<6) after 24 hours**	**40 (80%)**	**40 (80%)**	**NA**	**1.000**

* RA-ripening agent (PGE2)

**Table 4:** NICU admissions and stay in two groups

NICU	IMN (50)		Placebo (50)	
	No	%	No	%
**Admission**				
**Yes**	**1**	**2.0**	**3**	**6.0**
**No**	**49**	**98.0**	**47**	**94.0**
**No. of days stay**				
**2 days**	**0**	**0.0**	**1**	**2.0**
**3 days**	**1**	**2.0**	**1**	**2.0**
**5 days**	**0**	**0.0**	**1**	**2.0**

The mean Apgar scores at 1 min were 7.36± 1.35 in IMN group and 7.54± 1.01 in placebo group. The mean Apgar scores at 5 minutes were 8.70± 0.81 in IMN group and 8.78± 0.58 in placebo group.There were 5 babies in each of the IMN and placebo group who had Apgar scores less than 7 at 1 min. At 5 mins only 3 babies in IMN and 1 baby in placebo group had Apgar < 7. This difference in number of babies having Apgar scores< 7 at 5 minutes was statistically insignificant (p= 0.307).

3 babies from placebo group were admitted in NICU. One of them had Apgar score < 7 at 5 minutes. Only one baby from IMN group who had Apgar score < 7 at 5 minutes was admitted in NICU. The indication for admission for both groups was respiratory distress of newborn. The maximum neonatal stay was 5 days in one baby from the placebo group. The other 2 babies from placebo group stayed in NICU for 2 days and 3 days respectively. The one baby admitted in NICU from the IMN group stayed in NICU for 3 days for respiratory distress and was subsequently treated and sent back to the mother’s side (Table 4).

## Discussion

The need for induction of labour usually arises when the risks overweigh the benefits of continuing pregnancy. The condition or the favourability of cervix is an important prognostic factor in the success of induction of labour. Several studies are being done to identify the ideal cervical ripening agent. This study was conducted on 100 term pregnant women who were randomly divided into two groups: the isosorbidemononitrate (IMN) group and placebo group. The present study could not support our primary hypothesis that isosorbidemononitrate is more efficacious than placebo as a pre-induction cervical ripening agent. The results were comparable to other studies like Bollapragada et al. (4)who concluded that clinical utility of IMN as a pre-induction cervical ripening agent wasminimal. Though in their study, IMN had a significant effect on the change in cervical BishopScore (mean difference of 0.65, p = 0.013), they could not demonstrate asignificant change in their primary outcome measure which was admission to delivery interval (mean difference - 1.03 hrs, p = 0.68). In the present study, the baseline Bishop score was < 3 in all the patients similar to the study by Bollapragada et al. (4).

In our study, the mean difference in cervical scores at 24 hours was 0.01 with a p =0.983 and there was no significant change in drug insertion to delivery time. Osmanet al. (5) also concluded in their study that isosorbide mononitrate was less effective as a cervical ripening agent, though they used prostaglandins (PGE2) as the comparator. In contrast, Agarwal et al (6) attested to the effectiveness of IMN as cervical ripening agent by showing a significant change in Bishop scores after 2 doses of 40 mg IMN (3.17 ± 2.02; p < 0.001) and a reduction in admission to delivery interval (p < 0.001).This could have been because of inclusion of both nulliparous and multiparouswomen in their study. Levels of nitric oxide metabolites in cervix are more inmultiparous women which could explain the greater change seen in the Bishop scores. Further, there was no subgroup analysis based on parity regarding the difference in admission to delivery interval in their study. Habib et al. (7) also found significantly shorter admission to delivery intervals in the IMN group in contrast to placebo. This could be because of the differences in parity, gestational age at induction and the indication for induction. The mean gestational age at induction was 39.2 wks in their study as compared to 40.2 wks in ours. Further, only 35% were induced for postdates in their study whereas in the current study, such rate was 76%. Bothnulliparous and multiparous women were recruited in the afore-mentioned study byHabib et al. (7), whereas, we included only nulliparous women. They also did not conduct any subgroup analysis based on parity, and hence it is not possible to interpret whether their findings were applicable to both nulliparous and multiparouswomen or not. These differences could be attributed to the fact that nitric oxide metabolites are found to be reduced in amount in nulliparous women as well as in the cervical fluid of those delivering post-term as compared to those delivering near term, which could potentially contribute to the inability of the cervix to ripen in post-term pregnancies.

The proportion of women who went into labour in IMN and placebo groups in our study was 10% versus 4% respectively. Bollapragada et al. (4) did not report any differences in the number of women going into labour within the first 24 hrs (9% in IMN; 4% in placebo, p = 0.09). However, in the study conducted by Bullarboet al. (8), more women went into labour within 24 hrs in the IMN group than in theplacebo group. This can be explained by the differences in parity. These authors included both multiparous and nulliparous women in their study, as opposed to our study which included only nulliparous women. Subgroup analysis of this study showed that the proportions of primigravida women who had ripe cervix at the end of24 hrs was not significant, a result very similar to our observations (17% versus 8%, p= 0.11 in the study by Bullarbo et al. 10% versus 14% in our study).

Bollapragada et al. (4) showed a significant change in the proportion of women having unfavourable cervix after 48 hrs from drug initiation at home (64% in IMN vs77% in placebo, p = 0.02) which however was not reflected in our study (80% in each group, p = 1.000). This could have been because of the greater number of doses used in their study in comparison to ours (3 vs. 2 respectively) as well as due to the different time points of assessment (48 hrs in Bollapragada et al, 24 hrs in our study).

Almost equal number of women required a second ripening agent in our study (84% in IMN vs. 88% in placebo, p = 0.56), a result which was similar to that ofBollapragada et al. (4) (64% vs. 74%, p = 0.07).Most of the studies such as those conducted by Agrwal et al. (6) and Habib et al. (7), have shown lower incidence of caesarean deliveries in IMN group albeit statistically insignificant. Though the number of caesarean deliveries were more in IMN group than placebo in our study, it was not statistically significant. Most of these were done for fetal distress following PGE2 and failed induction of labour after 2 doses of PGE2and maximal doses of oxytocin infusion.

In the current study isosorbide mononitrate was found to be safe to use for cervical ripening without being associated with any adverse maternal and neonatal outcomes. There were no incidences of abnormal fetal heart rate patterns on CTG in the first 24 hrs, no differences in Apgar scores at 5 minutes and NICU admission rates. The results of neonatal outcome variables were in agreement with other studies conducted by Agarwal et al. (6) and Bollapragada et al. (4). Osman et al. (5) identified more number of abnormal fetal tracings in the PGE2 group (7%) as compared to IMNtherapy alone, and suggested that IMN may be safer to use as a cervical ripening agent in outpatient settings. Our study further showed that isosorbide mononitrate did not cause uterinehyper stimulation / tachysystole, nor did it result in any maternal side effects like headache, nausea, vomiting etc. Previous studies like Agarwal et al. (6), have also reported no incidences of tachysystole and hyper stimulation in the IMN group. There was no case of hyper stimulation in the IMN group in the study done by Habib et al. (7), but a higher incidence of tachysystole (1.96%) was observed in the IMN group. Most of the other studies have reported headache as the most common side effect of IMN,although it was treatable with simple analgesics like paracetamol. Maternalhemodynamics, like blood pressure or pulse rate was also not affected. In a study conducted by Ekerhovd et al. (9), significant changes were recorded in maternalhemodynamics which however did not prove to be of any clinical importance (at 180mins, change in maternal PR, p < 0.01, SBP, p < 0.001, DBP, p < 0.0001).

We noticed a few limitations in our study. We restricted our participants to only nulliparous women and did not evaluate the role of the drug in multiparous women. Majority of the women in our study were induced for post-dated pregnancy whose cervices have an inherent incapability to ripen and which could have resulted in the failure of the drug as a ripening agent. Use of a second ripening agent could have obscured our secondary outcome measures like mode of delivery where we saw a very high rate of operative delivery in the IMN group. Finally, we did not assess the maternal satisfaction with the use of the drug which is also an important determinant to judge successful outcome of induction of labour.

Based on the results and the methodology employed in the present study, we found that isosorbide mononitrate is an inefficient cervical ripening agent as it did not cause a significant change in cervical scores in comparison to placebo (mean difference at 24 hours -0.03, p= 0.983). Isosorbide mononitrate did not shorten the time interval from drug insertion to delivery when compared to placebo (mean difference -34.2 min, p= 0.52). However, there were no major adverse effects like abnormal fetal heart patterns or uterine hyper stimulation and tachysystole with the use of isosorbide mononitrate. It did not affect the maternal hemodynamics nor had any unfavourable neonatal outcomes which attest to its safety profile. To conclude, isosorbide mononitrate is a safe drug to use but we did not find it to be more efficient than a placebo to justify its clinical use as a cervical ripening agent. Further studies are required where the efficacy of nitric oxide donors for cervical ripening using different formulations, doses and in combination with other ripening agents may be evaluated.
